# Combined tazemetostat and MAPKi enhances differentiation of papillary thyroid cancer cells harbouring *BRAF* ^V600E^ by synergistically decreasing global trimethylation of H3K27

**DOI:** 10.1111/jcmm.15007

**Published:** 2020-01-22

**Authors:** Hao Fu, Lin Cheng, Ri Sa, Yuchen Jin, Libo Chen

**Affiliations:** ^1^ Department of Nuclear Medicine Shanghai Jiao Tong University Affiliated Sixth People’s Hospital Shanghai China

**Keywords:** differentiation therapy, EZH2 inhibitor, H3K27me3, MAPK inhibitor, thyroid cancer

## Abstract

Clinical efficacy of differentiation therapy with mitogen‐activated protein kinase inhibitors (MAPKi) for lethal radioiodine‐refractory papillary thyroid cancer (RR‐PTC) urgently needs to be improved and the aberrant trimethylation of histone H3 lysine 27 (H3K27) plays a vital role in *BRAF* ^V600E^‐MAPK‐induced cancer dedifferentiation and drug resistance. Therefore, dual inhibition of MAPK and histone methyltransferase (EZH2) may produce more favourable treatment effects. In this study, *BRAF* ^V600E^‐mutant (BCPAP and K1) and *BRAF*‐wild‐type (TPC‐1) PTC cells were treated with MAPKi (dabrafenib or selumetinib) or EZH2 inhibitor (tazemetostat), or in combination, and the expression of iodine‐metabolizing genes, radioiodine uptake, and toxicity were tested. We found that tazemetostat alone slightly increased iodine‐metabolizing gene expression and promoted radioiodine uptake and toxicity, irrespective of the *BRAF* status. However, MAPKi induced these effects preferentially in *BRAF* ^V600E^ mutant cells, which was robustly strengthened by tazemetostat incorporation. Mechanically, MAPKi‐induced decrease of trimethylation of H3K27 was evidently intensified by tazemetostat in *BRAF* ^V600E^‐mutant cells. In conclusion, tazemetostat combined with MAPKi enhances differentiation of PTC cells harbouring *BRAF* ^V600E^ through synergistically decreasing global trimethylation of H3K27, representing a novel differentiation strategy.

## 1 INTRODUCTION

True increases in the global occurrence of thyroid cancer have been reported recently, owing to a steady rise in the incidence of papillary thyroid cancer (PTC), which accounts for approximately 85% of all thyroid cancers.^1^, ^2^, ^3^ ^131^I therapy is a conventional and effective treatment for unresectable disease, which is based on the nature of radioiodine avidity of tumour originating from thyroid follicular cells via the sodium/iodide symporter (NIS). However, nearly half of the persistent/recurrent or metastatic lesions lose the ability to take up radioiodine de novo or go through a dedifferentiation process, which is named radioiodine‐refractory papillary thyroid cancer (RR‐PTC). A major mechanism underlying the development of RR‐PTC is the aberrant silencing of iodine‐metabolizing genes such as *Nis*, *Tshr*, *Tg* and *Tpo*, which is a result of *BRAF* ^V600E^ mutation‐induced activation of the mitogen‐activated protein kinase (MAPK) pathway.^4^

Increasing evidences, including our previous study, have demonstrated that MAPK inhibitors (MAPKi) can induce differentiation in thyroid cancer^5^, ^6^; however, their clinical effectiveness in restoring ^131^I uptake remains insufficient.^7^, ^8^, ^9^, ^10^ Thought‐provokingly, reinduction of ^131^I uptake in DTC patients treated with sorafenib failed even in vitro study had showed promising data.^7^ Besides, the *BRAF* ^V600E^ inhibitor dabrafenib restored new radioiodine uptake in 60% of 10 patients, but the objective response rate was only 20%.^8^ In addition, although the clinically tested MEK inhibitor selumetinib had shown promising differentiation efficacy, it unfortunately seemed to be less effective in thyroid cancers with a *BRAF* mutation.^9^ Hence, the differentiation effect of MAPK inhibitors remains to be improved, so that ^131^I may sufficiently exert its theranostics actions.

It is well known that histone H3 lysine 27 (H3K27) trimethylation modification (H3K27me3) leads to depression of gene expression through enhancer of zeste homolog 2 (EZH2), a critical methyltransferase catalysing H3K27 and an epigenetic mark for the maintenance of gene silencing.^11^ More recently, hyper‐trimethylation on H3K27 has been demonstrated to be associated with cancer cell dedifferentiation and resistance to BRAF inhibitor treatment.^12^ Additionally, EZH2 has been clinically found to express in poorly differentiated and anaplastic thyroid cancers, correlating with poorer survival,^13^ and H3K27me3 expression was up‐regulated particularly in thyroid cancer with aggressiveness phenotype and associated with dedifferentiation of thyroid cancer.^14^ Therefore, inhibiting the activity of EZH2 by specific inhibitors represents a potential direction of differentiation therapy.

Furthermore, MAPK signal aberrant activation by *BRAF* ^V600E^ has also been demonstrated to increase the level of H3K27me3 through increasing the expression of *Ezh2* in thyroid cancer.^15^ Conversely, the decrease of H3K27me3 via reducing the expression of EZH2 by MAPKi was fulfilled in thyroid cancer, and the differentiation markers in melanoma and neuroblastoma could be increased by EZH2 knockdown.^12^, ^15^, ^16^ However, the differentiation efficacy of EZH2 inhibitor alone or combined with MAPKi in thyroid cancer remains unknown. We, therefore, conceived this study to evaluate the differentiation efficacy of EZH2 inhibitor, assess the impact on differentiation induced by EZH2 inhibitor combined with MAPKi and elucidate the underlying mechanisms in PTC cell lines.

## 2 MATERIALS AND METHODS

### 2.1 Agents and cell culture

According to the identification findings of all PTC cell lines globally available, the *BRAF* ^V600E^‐mutant cell lines (BCPAP, K1) and the wild‐type *BRAF* cell line (TPC‐1) were used.^17^ The BCPAP and TPC‐1 cell lines were purchased from the Chinese Academy of Science, and the K1 cell line was obtained from the Health Protection Agency culture collection. Nthy‐ori 3‐1, a normal thyroid follicular epithelial cell line immortalized by SV‐40, was obtained from the European Collection of Cell Cultures (Wiltshire, United Kingdom).^18^ All cells were cultured in RPMI 1640 medium with 10% foetal bovine serum at 37°C and 5% CO_2_. Regarding findings of pre‐experiments, concentrations of MAPKi were set as dabrafenib (MCE) at 0.1 μM, selumetinib (MCE) at 4 μM and tazemetostat, the EZH2 inhibitor EPZ6438 (MCE), at 1 μM, which were found to induce preferable differentiation effects. Such concentrations were used individually or in combination for the indicated time intervals in the following experiments. All the cells were incubated overnight before treated with the medicines. Dimethyl sulfoxide (DMSO, 0.05 mM; Sigma) was used in parallel as the vehicle control. After the first 24 hours treatment with the indicated inhibitors, bovine thyroid‐stimulating hormone (TSH; Millipore) at 1 mU/mL was added for an additional 24/48 hours to stimulate the expression of thyroid‐specific genes or ^125^I uptake.

### 2.2 RNA extraction and real‐time qRT‐PCR analysis

Cells (2.0 × 10^5^) were seeded in 9.6 cm^2^ plates and then treated with MAPKi (dabrafenib/selumetinib) or tazemetostat individually or in combination, or with DMSO. Total RNA was isolated from cells using the RNA‐Quick Purification Kit (Yishan), Total RNA (1 µg) was converted to cDNA on an ABI Veriti™ 96‐Well Thermal Cycler (Thermo Fisher) using HiScript II Q RT SuperMix for qPCR (Vazyme). Real‐time quantitative RT‐PCR analysis was performed on an Applied Biosystems 7500 Real‐Time PCR Systems (Applied Biosystems) using AceQ qPCR SYBR Green Master Mix (Vazyme). *β‐Actin* was run in parallel to standardize the input cDNA. The primers designed for thyroid‐specific genes and the methods used to calculate relative expression levels of these genes were as described previously.^19^

### 2.3 Western blotting assay

Histones were extracted from cells according to the instruction of Histone Extraction Kit (Abcam). For whole‐cell lysates, cells were lysed in RIPA buffer. Equal amounts of total protein were resolved by SDS‐PAGE, transferred to PVDF membranes (Millipore) and immunoblotted with the indicated primary antibodies. Membranes were hybridized with the following primary antibodies: p‐Erk1/2, Erk1/2, EZH2, H3K27me3 (Cell Signaling Technology), c‐Myc, H3 (Abcam), NIS, Tg (thyroglobulin), TPO (thyroid peroxidase), TSHR and GAPDH (Protein tech), all the antibodies were diluted at 1:1000. Membranes were then hybridized with species‐specific HRP‐conjugated antibodies (1:5000; Cell Signaling Technology). Bands were visualized using the Potent ECL kit (Yeasen).

### 2.4 Immunofluorescent localization of NIS

Cells (2.0 × 10^4^) were seeded in six‐well chamber slides. After 72 hours of incubation with specific inhibitors, cells were fixed in paraformaldehyde and blocked with 1% BSA. Cells were then incubated in succession with rabbit anti‐NIS (1:100; Protein tech), and Goat Anti‐Rabbit IgG H&L (FITC) (Abcam) diluted at 1:100, and DAPI. NIS expression was monitored by fluorescent microscopic examination (Leica SP8, Germany).

### 2.5 ^125^I uptake assay

Cells (1.5 × 10^5^) were seeded in six‐well plates and then incubated with MAPKi and tazemetostat individually, or in combination, or with DMSO for 72 hours. ^125^I uptake assay was performed as previously described by our team.^20^ Briefly, one well was counted for cell number for each group, and the remaining wells were incubated in 1 mL serum‐free RPMI 1640 containing 74 kBq Na^125^I at 37°C for 1 hour. The medium containing Na^125^I was then removed, and the cells were washed twice with PBS and lysed with 0.3 M sodium hydroxide on ice. The radioactivity in cell lysates was measured with a gamma counter.

### 2.6 In vitro clonogenic assay

Cells (4 × 10^2^) were seeded into six‐well plates and cultivated overnight to allow attachment. After 72 hours treatment with targeted agents, a clonogenic assay was performed as previously described with minor modifications.^20^ Briefly, drug containing medium was discarded and cells were washed twice with PBS. The medium was then replaced with 1 mL of regular culture medium in the presence or absence of 20 μCi Na^131^I for 6 hours. The radioactive medium was discarded at the end of the treatment, and cells were incubated in regular culture medium for 7 days. Finally, cells were fixed in methanol and stained with crystal violet and the number of macroscopic colonies was counted.

### 2.7 Statistical analysis

All the experiments were carried out at least three times. The data from the RT‐PCR assay and ^125^I uptake were compared using the independent‐samples *t* test. All statistical analyses were performed using a statistical software program (SPSS, version 20.0; SPSS, Inc). Significance was defined as *P* < .05.

## 3 RESULTS

### 3.1 Tazemetostat with associated MAPKi enhances transcription of iodide‐handling genes

**Figure 1:**
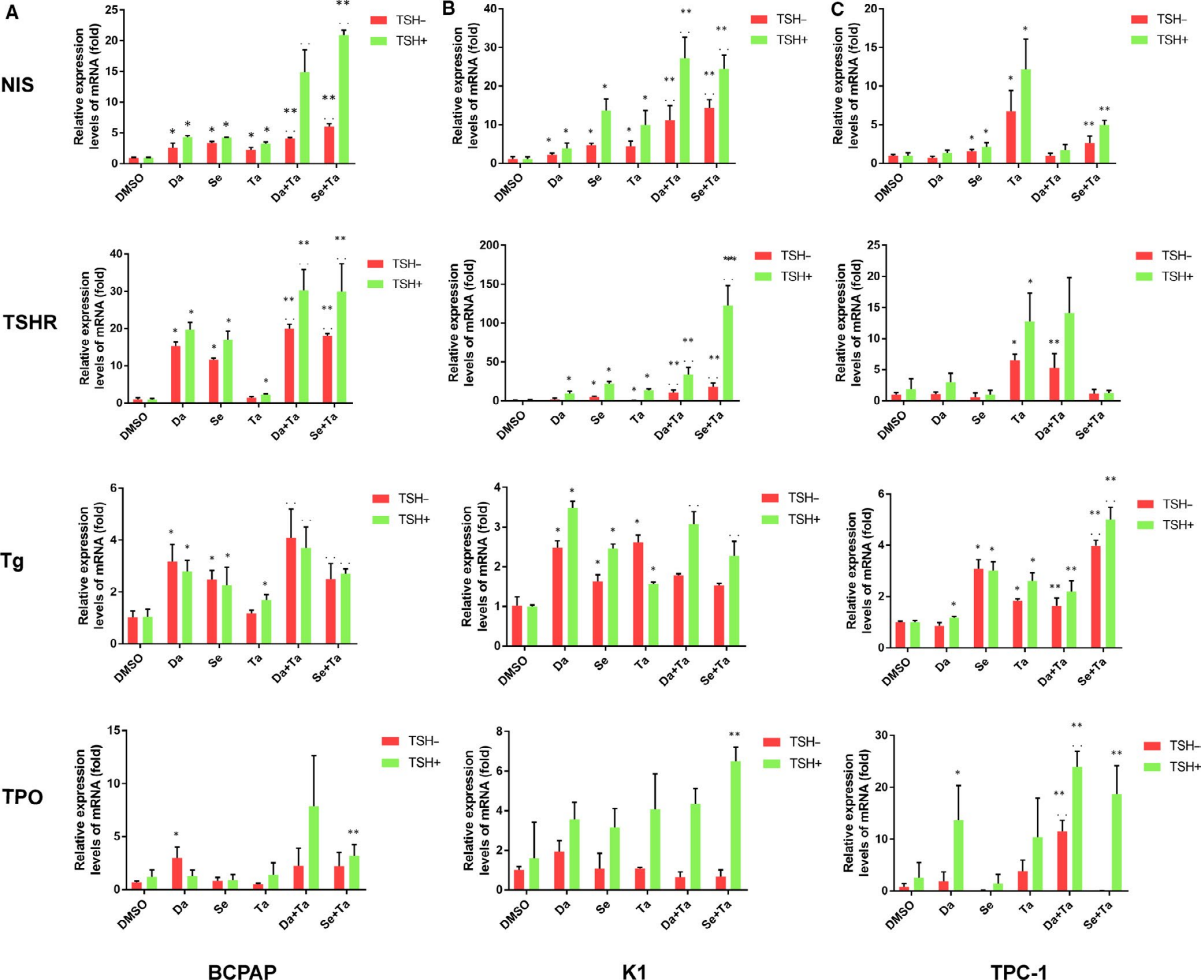
Effects of MAPKi (dabrafenib/selumetinib) and tazemetostat alone or in combination on the transcriptions of iodide‐handling genes (*Nis*, *Tshr*, *Tg*, *Tpo*) in BCPAP (A), K1 (B), and TPC‐1 (C) cells. Data are presented as means ± SD. *P < .05 for comparison with DMSO control. **P < .05 for comparison with the MAPKi‐treated group.**^.^** *P* < .05 for comparison with the tazemetostat‐treated group. *Nis*, sodium iodine symporter; *Tshr*, thyroid‐stimulating hormone receptor; *Tg*, thyroglobulin; *Tpo*, thyroid peroxidase; Da, dabrafenib; Se, selumetinib; Ta, tazemetostat

The mRNA levels of *Nis*, *Tshr* and *Tg* were restored in K1 and TPC‐1 cells treated with tazemetostat alone (*P* < .05), whereas only the mRNA level of *Nis* was promoted in BCPAP treated with tazemetostat (*P* < .05). Even less, the expression of *Tpo* remained unchanged in all the three PTC cell lines treated with tazemetostat (Figure 1).

When treated with MAPKi (dabrafenib or selumetinib) alone, compared with DMSO treatment groups, up‐regulated transcriptions could be identified in BCPAP cells (*Nis*, *Tshr* and *Tg*) and K1 cells (*Nis* and *Tg*), whereas the expression of *Tpo* enhanced only in dabrafenib‐treated BCPAP cells (*P* < .05). Additionally, the expression of *Tshr* in K1 cells and both *Nis* and *Tg* in TPC‐1 cells were promoted when treated with selumetinib, respectively (*P* < .05). In TPC‐1 cells, compared with DMSO‐treated control, dabrafenib did not increase transcription of iodide‐handling genes (*P* > .05) (Figure 1).

Notably, compared with the tazemetostat‐treated groups or MAPKi‐treated groups, the combination of tazemetostat with MAPKi induced a more robust expression of *Nis* and *Tshr* only in BCPAP and K1 cells (*P* < .05). Interestingly, the combined regime of tazemetostat and dabrafenib elevated the expression of *Tpo* in TPC‐1 cells when compared with the tazemetostat‐treated groups or dabrafenib‐treated groups. The expression of *Tg* in TPC‐1 cells treated with selumetinib plus tazemetostat was significantly higher than that in the tazemetostat‐treated groups or selumetinib‐treated group (*P* < .05) (Figure 1).

We subsequently investigated whether TSH could further stimulate the expression of thyroid genes in thyroid cancer cells since TSHR plays an important role in up‐regulating iodide‐handling genes in thyroid cells. After treatment with MAPKi or tazemetostat or in combination for 24 hours, TSH was added to further treat the cells for another 24 hours. As shown in Figure 1, TSH dramatically enhanced the effects of MAPKi or tazemetostat or combined therapies on the expression of all thyroid genes involved in the three PTC cell lines, including its own receptor gene *Tshr* (*P* < .05).

### 3.2 Tazemetostat with concomitant MAPKi improves expression of iodide‐handling proteins

**Figure 2:**
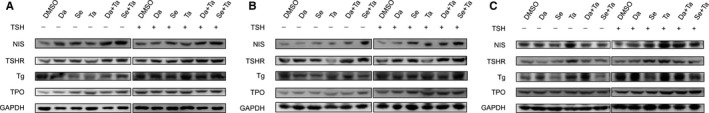
Western blotting demonstrating the effects of various treatments on the protein levels of NIS, TSHR, Tg and TPO in BCPAP (A), K1 (B) and TPC‐1 (C) cells. GAPDH was used as positive control. NIS, sodium iodine symporter; TSHR, thyroid‐stimulating hormone receptor; Tg, thyroglobulin; TPO, thyroid peroxidase; Da, dabrafenib; Se, selumetinib; Ta, tazemetostat; GAPDH, glyceraldehyde‐3‐phosphate dehydrogenase

As shown in Figure 2, NIS, TSHR and Tg expression were distinctly increased in TPC‐1 cells treated with tazemetostat, but only mildly restored in BCPAP and K1 cells. Moreover, NIS and TSHR were enhanced in BCPAP and K1 cells treated with MAPKi, whereas Tg was not obviously changed. Additionally, slightly enhanced expression of NIS was observed in TPC‐1 cells treated with selumetinib. Dabrafenib exerted no effect on the expression of iodide‐handling proteins in TPC‐1 cells. Apparently, compared with the tazemetostat‐treated groups or the MAPKi‐treated groups, fiercely elevated expression of NIS and TSHR was observed only in BCPAP and K1 cells when the three cell lines were treated with the combination of tazemetostat with MAPKi. Undoubtedly, the protein expression of thyroid genes was enhanced in varying degrees after TSH incorporation. Nevertheless, no significant change of TPO could be visualized in three cell lines when treated with MAPKi or tazemetostat or in combination.

**Figure 3:**
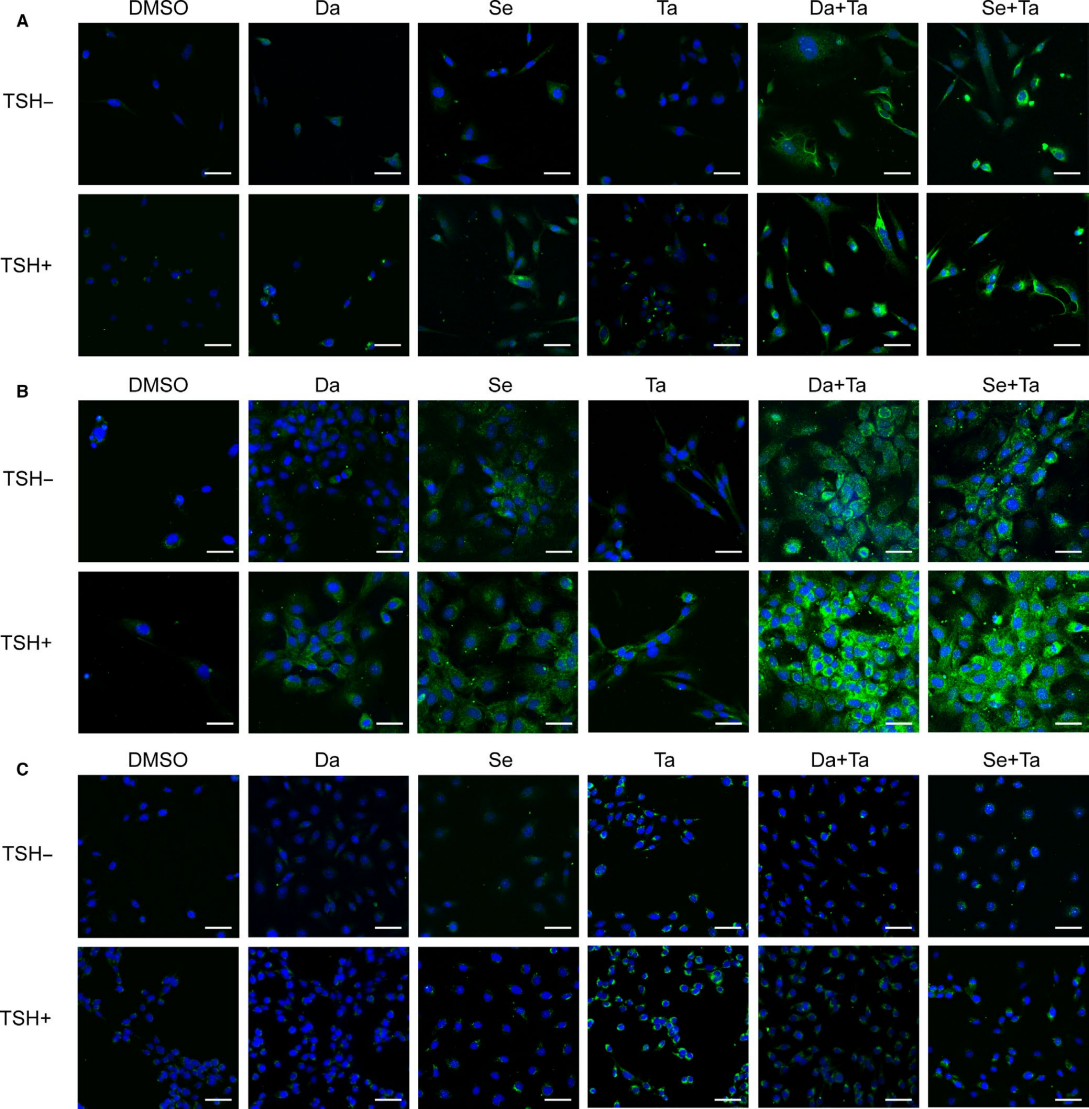
Immunofluorescent microscopic analysis of NIS protein expression. *BRAF* ^V600E^‐mutant (A, BCPAP; B, K1) and *BRAF*‐wild‐type (C, TPC‐1) cells were treated with 0.1 μM dabrafenib/4 μM selumetinib or 1 μM tazemetostat alone or in combination. The blue colour represents DAPI nuclear staining, and the green colour represents NIS staining. Scale bars represent 25 μm in all panels. Da, dabrafenib; Se, selumetinib; Ta, tazemetostat

As NIS plays the foremost role in thyroid cell iodide uptake, we further examined NIS protein expression by immunofluorescent microscopy. As shown in Figure 3, there was virtually impalpable NIS protein expression when treated with DMSO. NIS expression was up‐regulated to varying degrees by tazemetostat in the three PTC cell lines and by MAPKi only in BCPAP and K1 cells, which was prominently seen in the cytoplasm and mildly promoted by TSH. Of note, robust expression of NIS protein and clear localization in cell membrane were achieved by dual inhibition with MAPKi and tazemetostat merely in BCPAP and K1 cells, which were also intensified by TSH.

### 3.3 Tazemetostat with combined MAPKi synergistically induces de‐trimethylation of H3K27

**Figure 4:**
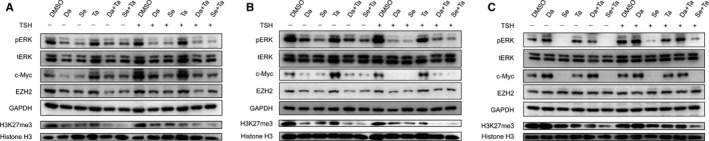
Western blotting of lysates and histone extraction of BCPAP (A), K1 (B) and TPC‐1 (C) demonstrating the effects of various treatments on the protein levels of MAPK, c‐Myc, EZH2 and H3K27me3. DMSO was used as the vehicle control. Da, dabrafenib; Se, selumetinib; Ta, tazemetostat

As shown in Figure 4, the phosphorylation of ERK (p‐ERK) was preferentially inhibited in BCPAP and K1 treated with dabrafenib/selumetinib, which further decreased the expression of c‐Myc and EZH2 and ultimately reduced the effector protein of EZH2, H3K27me3. In TPC‐1 cells, however, the p‐ERK was decreased only in selumetinib treatment, which was followed by down‐regulation of c‐Myc. Unlike the phenomenon observed in BCPAP and K1 cells, the expression of both EZH2 and H3K27me3 was not significantly decreased in TPC‐1 treated with MAPKi. Although tazemetostat did not decrease expression of EZH2, it specifically inhibited the activity of EZH2 and reduced H3K27me3 in all the cell lines, irrespective of the *BRAF* status. In BCPAP and K1 cells, H3K27me3 was further decreased when cells were treated with dual inhibition with MAPKi and tazemetostat compared with MAPKi or tazemetostat treatment alone. Likewise, in TPC‐1 cells, H3K27me3 was decreased by combination of selumetinib with tazemetostat compared with the selumetinib or tazemetostat treatment alone, although H3K27me3 in group of dabrafenib combined with tazemetostat was decreased when compared to dabrafenib treatment alone, it was not changed obviously when compared to that in tazemetostat treatment alone. It is worth mentioning that the addition of TSH did not significantly change the expression of these proteins (Figure 4).

### 3.4 Tazemetostat with concurrent MAPKi promotes ^125^I uptake

**Figure 5:**
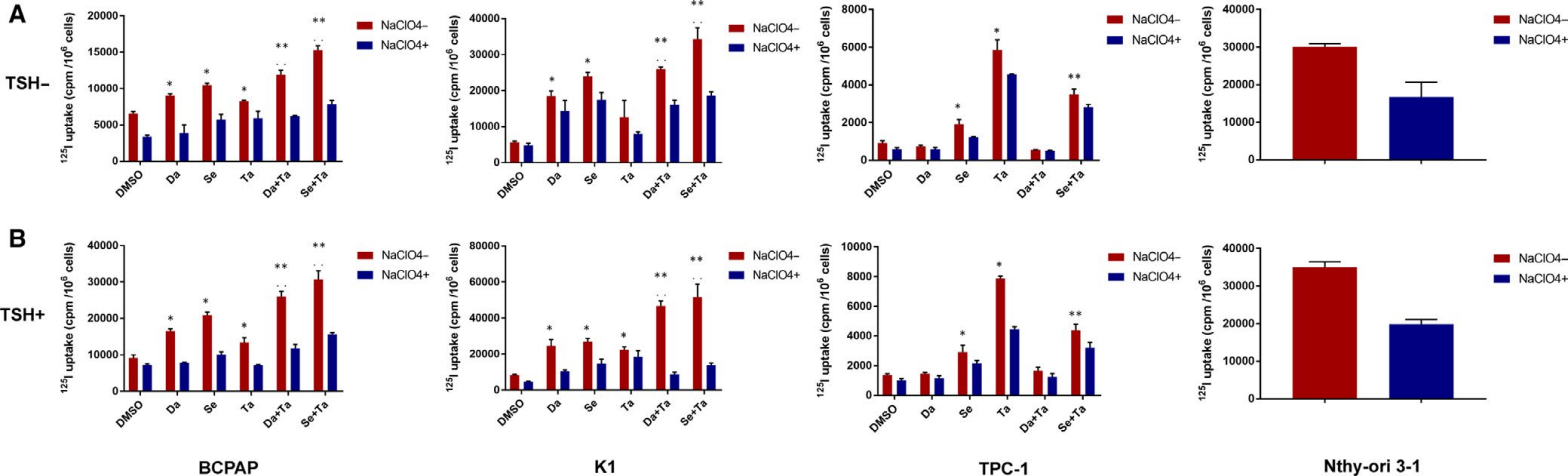
Radioactive iodine (^125^I) uptake in BCPAP, K1, TPC‐1 cells and Nthy‐ori 3‐1. A, TSH (−); B, TSH (+). Data were expressed as means ± SD. *P < .05 for comparison with DMSO‐treated cells. **P < .05 for comparison with the MAPKi‐treated group.**^..^** *P* < .05 for comparison with the tazemetostat‐treated group. Da, dabrafenib; Se, selumetinib; Ta, tazemetostat; TSH, thyroid‐stimulating hormone

Compared with DMSO‐treated cells, ^125^I uptake was 1.26‐fold higher in BCPAP cells (*P* < .05), 2.23‐fold higher in K1 cells, and 5.90‐fold higher in TPC‐1 cells (*P* < .05) treated with tazemetostat (Figure 5). When incubated with dabrafenib, ^125^I uptake was 1.39‐fold higher in BCPAP cells (*P* < .05) and 3.28‐fold higher in K1 cells (*P* < .05) compared with non‐treated groups, and there was no evident change in iodine uptake in TPC‐1 cells. When treated with selumetinib, ^125^I uptake was 1.60‐fold and 4.24‐fold higher in BCPAP and K1 cells, respectively (*P* < .05), and a 2.07‐fold higher ^125^I uptake was also found in TPC‐1 cells (*P* < .05). In BCPAP and K1 cells, simultaneous suppression of EZH2 and BRAF induced 1.82‐fold and 4.61‐fold higher ^125^I uptake, respectively (*P* < .05); dual suppression of EZH2 and MEK induced 2.34‐fold and 6.07‐fold higher ^125^I uptake, respectively (*P* < .05). In TPC‐1 cells, compared with DMSO‐treated control, the combination of dabrafenib and tazemetostat did not induce any ^125^I‐uptake (*P* > .05), but the combination of selumetinib and tazemetostat‐induced elevated ^125^I‐uptake (*P* < .05). Encouragingly, the degrees of ^125^I uptake in K1 cells treated with combined therapies were high as that of Nthy‐ori 3‐1 (Figure 5).

Besides, in comparison with dabrafenib or selumetinib, the combination of dabrafenib or selumetinib with tazemetostat induced 1.31‐fold (*P* < .05) and 2.34‐fold (*P* < .05) higher ^125^I uptake in BCPAP cells, respectively; 1.41‐fold (*P* < .05) and 1.43‐fold (*P* < .05) in K1 cells, respectively. Additionally, a 1.83‐fold (*P* < .05) higher ^125^I uptake was observed TPC‐1 cells when tazemetostat was simultaneously added compared with selumetinib treatment alone, whereas dual inhibition with tazemetostat and dabrafenib produced no effect compared with dabrafenib treatment alone. Likewise, in comparison with tazemetostat, the combination of tazemetostat with dabrafenib or selumetinib induced 1.44‐fold (*P* < .05) and 1.85‐fold (*P* < .05) higher ^125^I uptake in BCPAP cells respectively; 2.07‐fold (*P* < .05) and 2.72‐fold (*P* < .05) in K1 cells, respectively. However, the combination of tazemetostat with MAPKi does no elevate ^125^I uptake when compared to tazemetostat treatment alone in TPC‐1 cells (Figure 5).

Thyroid‐stimulating hormone enhanced ^125^I uptake on the basis of these inhibitors, while TSH alone had only a minimal effect on ^125^I uptake. The most robust magnitude of ^125^I uptake was seen with the triple combination of tazemetostat, MAPKi, and TSH in cells harbouring the *BRAF* ^V600E^ mutation, especially in K1 cells, which was ever higher than that of Nthy‐ori 3‐1. Furthermore, the increased ^125^I accumulation in the three PTC cell lines treated with these inhibitors could be reduced by sodium perchlorate (Figure 5).

### 3.5 Tazemetostat with associated MAPKi elevates radioiodine toxicity

**Figure 6:**
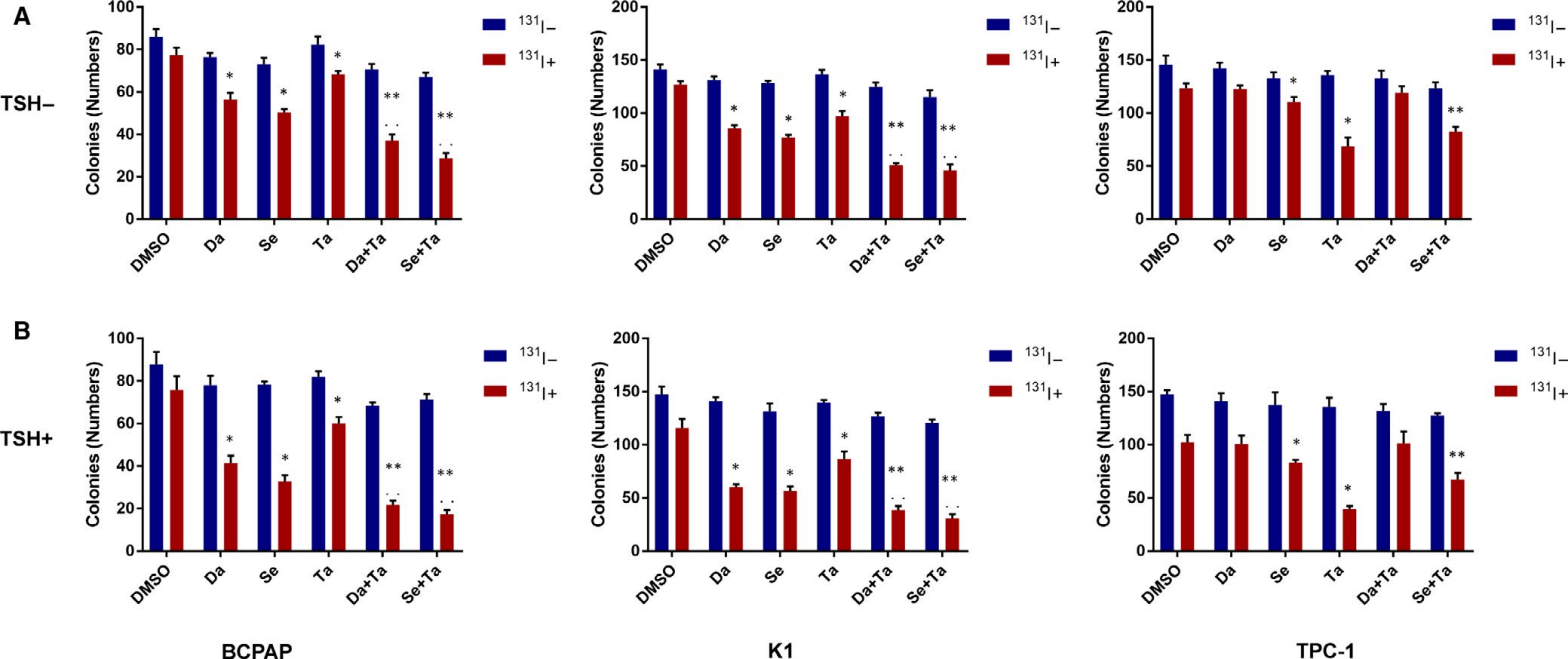
In vitro clonogenic assay. A, TSH (−); B, TSH (+). Data were expressed as means ± SD. *P < .05 for comparison with DMSO‐treated cells. **P < .05 for comparison with the MAPKi‐treated group. *P* < .05 for comparison with the tazemetostat‐treated group. Da, dabrafenib; Se, selumetinib; Ta, tazemetostat; TSH, thyroid‐stimulating hormone

The numbers of colonies decreased significantly after ^131^I treatment in both BCPAP and K1 cells pretreated with MAPKi or tazemetostat alone (*P* < .05) compared with DMSO‐pretreated control (Figure 6). Compared with MAPKi‐pretreated or tazemetostat‐pretreated groups, the numbers of colonies further reduced when tazemetostat and MAPKi were incorporated (*P* < .05).

In TPC‐1 cells, dabrafenib did not induce any ^131^I‐toxicity, even when coupled with tazemetostat (*P* > .05). The numbers of colonies mildly reduced after ^131^I treatment when pretreated with selumetinib and evidently decreased after ^131^I treatment when pretreated with tazemetostat (*P* < .05). Moreover, the numbers of colonies significantly reduced when treated with a combination of selumetinib with tazemetostat compared with selumetinib treatment alone (*P* < .05), but were not less than that in tazemetostat treatment group. TSH alone had a negligible impact on ^131^I toxicity, whereas it amplified the ^131^I toxicity induced by the above various treatments.

## 4 DISCUSSION

RR‐PTC represents a major therapeutic challenge in thyroid cancer medicine, which is mainly caused by *BRAF* ^V600E^ mutation that leads to the abnormal activation of MAPK pathway.^21^, ^22^ Recent evidences have indicated that oncogenic signalling controls several key processes of epigenetic gene regulation.^23^ Increasing numbers of studies, including ours, have confirmed that tumour epigenetics‐based therapeutics offer a new direction for differentiation therapy.^19^, ^24^, ^25^ Kong et al found that histone hypermethylation resulted in cancer cell dedifferentiation and resistance to BRAF inhibitor treatment, which was largely mediated by H3K27me3. They further demonstrated that knockdown of the H3K27‐specific methyltransferase EZH2 attenuated the H3K27me3‐mediated effects in vitro and in vivo.^12^ In addition, H3K27me3 overexpression is associated with aggressiveness and dedifferentiation of thyroid cancer.^14^ Furthermore, Hou and colleagues have elucidated that *BRAF* ^V600E^ regulates the expression of *Ezh2*, *Suz12* and *Jarid2* by c‐Myc, resulting in changes in H3K27me3 and subsequent epigenetic silencing. EZH2 is a critical methyltransferase catalysing H3K27 and exhibits inappropriate expression in various aggressive cancers including thyroid carcinoma.^26^, ^27^ Tazemetostat, a novel EZH2 inhibitor, has showed a favourable antitumour activity and represented a potential agent for sorafenib‐resistant thyroid carcinoma.^28^, ^29^

The present study provides a new horizon in restoring treatment of RR‐PTC. A minimal differentiation effect of MAPKi with *BRAF* ^V600E^ preferentiality is consistent with previous findings.^6^, ^9^, ^19^ However, a slight but significant differentiation effect without *BRAF* ^V600E^ dependence by tazemetostat per se was firstly identified by our group. More meaningfully, the differentiation efficacy induced by tazemetostat incorporated with MAPKi was strikingly enhanced in *BRAF* ^V600E^‐mutant PTC cell lines, indicating that simultaneous suppression of EZH2 in both amount and activity using combination of MAPKi with tazemetostat could synergestically promote differentiation in PTC cells harbouring *BRAF* ^V600E^.

Encouraged by enhancement of transcription and translation of *Nis* and *Tshr* induced by tazemetostat combined with MAPKi, immunofluorescence, radioiodine uptake and toxicity were explored to display NIS protein localization and virtual function, which surpassed previous differentiation therapy studies limited to the mRNA and protein levels.^30^, ^31^ In line with previous findings, tazemetostat promoted ^125^I uptake in PTC cell lines irrespective of *BRAF* status, whereas MAPKi‐induced ^125^I uptake mainly in *BRAF* ^V600E^‐positive cell lines, which substantially increased when coupled with tazemetostat. In addition, sodium perchlorate inhibited the accumulation of ^125^I, which means that increased uptake is NIS‐specific. Furthermore, the in vitro clonogenic assay also demonstrated that tazemetostat combined with MAPKi enhanced ^131^I toxicity in *BRAF* ^V600E^‐mutant PTC cells. Overall, the patterns of ^125^I uptake and ^131^I toxicity indicate that tazemetostat associated with MAPKi markedly enhances thyroid cancer cell differentiation in *BRAF* ^V600E^‐positive cells.

*Nis*, *Tshr*, *Tg* and *Tpo* are the genes involved in thyroid hormone biosynthesis, regulating the uptake and organification of iodine, and considered to be the key markers of differentiation.^32^ It should be noted that the expression of *Tg* and *Tpo* was not increased as much as that of *Nis* and *Tshr*, and there was no evident differential expression of *Tg* and *Tpo* at the protein level among various treatments. These phenomena have also been found in the study by Xing et al,^33^ indicating that other factors may be involved in the regulation of the expression of *Tg* and *Tpo*.^34^ Moreover, TPO was not obviously elevated in our study, which may compromise the final lethal effect of radioiodine on tumour cells, due to that iodide anion not organized by TPO undergoes rapidly efflux from cells, and a balance between NIS‐mediated iodide uptake and TPO‐mediated efflux determines the intracellular concentration and radiation dose of radioiodine.^35^

Conversely, both iodine uptake and toxicity assays consistently showed that differentiation effect of the combination of selumetinib with tazemetostat was more intensive than that of selumetinib monotherapy in *BRAF*‐wild‐type cell line, but subtler than that of tazemetostat monotherapy. Furthermore, the differentiation effect of the combination of dabrafenib with tazemetostat was comparable to that of dabrafenib monotherapy in *BRAF*‐wild‐type cell line, but much lower than that of tazemetostat monotherapy. These complementary data derived from the designed control suggest that the synergistic differentiation role of might be *BRAF* ^V600E^ dependent.

In the study of underlying mechanism of pathogenesis of thyroid cancer, Hou et al have elucidated that the increase of H3K27me3 induced by *BRAF* ^V600E^ mutation was achieved by enhancing the expression of c‐Myc followed by the expression of EZH2.^15^ Besides, tazemetostat‐induced differentiation has also been carried out by reducing H3K27me3 via directly inhibiting EZH2 activity in neuroblastoma.^16^ Therefore, the combination of MAPKi with tazemetostat in our study not only reduced the expression of EZH2 but inhibited its activity as well, yielding robust reduction of the downstream H3K27me3, which effectively helped to enhance the differentiation of *BRAF* ^V600E^‐mutant PTC cells. In control cell line (TPC‐1), H3K27me3 was decreased when tazemetostat was coupled with MAPKi compared with MAPKi monotherapy, but dabrafenib did not intensify the impact of tazemetostat on H3K27me3, hinting a tazemetostat‐predominant effect. However, H3K27me3 was seemingly decreased in the selumetinib plus tazemetostat‐treated group compared with tazemetostat‐treated group, in which the underlying mechanism needs to be further investigated, since the expression of EZH2 remained stable irrespective of treatments in TPC‐1 cells.

TSH is a master regulator in up‐regulating the iodide‐handling machinery in thyroid cells through activating the TSHR.^36^ In the present study, expression of TSHR was also robustly induced by dual suppression with MAPK and EZH2 inhibitors, which could be intensified by the incorporation of TSH. Our study demonstrated that TSH merely amplified the thyroid gene expression and their activities with the aid of MAPKi or tazemetostat or in combinations, as TSH alone virtually exerted no effect on thyroid gene expression. Additionally, TSH also promoted NIS localization to the cell membrane, a critical step for NIS‐mediated radioiodine uptake into cells. These results provide a reliable basis for the elevation of serum TSH via recombinant human TSH injection or thyroxine withdrawal to enhance radioiodine avidity of PTC.^37^

There are some limitations in the current study. Firstly, this is an in vitro study, and our findings need to be verified by in vivo studies for translation from bench to beside. Secondly, although it has been demonstrated that synergistic inhibition of EZH2 simultaneously using MAPK and EZH2 inhibitors intensively suppressed the trimethylation of H3K27 and ultimately enhanced iodine‐avidity in *BRAF* ^V600E^‐mutant PTC cells, the concrete underlying mechanism of up‐regulation of iodine‐handling genes by decrease of H3K27me3 needs further investigation.

In conclusion, this study demonstrated that tazemetostat combined with MAPKi may effectively enhance differentiation of PTC cells harbouring *BRAF* ^V600E^ via synergistically decreasing global trimethylation of H3K27, which may be potentially translated into a novel differentiation therapeutic strategy.

## CONFLICT OF INTEREST

The authors declare that they have no competing interests.

## AUTHOR CONTRIBUTIONS

Hao Fu and Lin Cheng conducted the experiments and wrote the paper. Ri Sa and Yuchen Jin worked on figures and edited the manuscript. Libo Chen conceived the idea, designed the experiments and gave final approval of the version to be published.
